# Actual and Future Employment for Radiologists in Belgium: Results of a Survey

**DOI:** 10.5334/jbsr.1756

**Published:** 2019-05-22

**Authors:** Matthias Lavens, Barbara Geeroms, Cedric Bohyn

**Affiliations:** 1 UZ leuven, BE

**Keywords:** Job employment, radiology departments, opportunities for radiologists, survey

## Abstract

**Introduction:**

Belgium counts 1,888 active radiologists. This is an average of 16.2 radiologists per 100,000 people, which is slightly more than the European average of 12.7 per 100,000. Feedback from recently graduated residents suggests difficulties in finding a permanent staff member position and a high demand for dedicated profiles in radiology departments. To objectify this, the Young Radiologist Section (YRS) of the Belgian Society of Radiology (BSR) performed a survey of the radiology job market in Belgium.

**Material and Methods:**

An anonymous survey was sent to recently graduated Belgian radiologists (2013–2018) and to the heads of all Belgian radiology departments.

**Results:**

The majority of the responding graduates found a permanent staff member position as a radiologist within two years after graduation and around half of the respondents even before graduation (50% in the graduates 2018 and 57% in graduates of 2013–2017). However, a small portion of the responding graduates (8%) needed more than two years to find a staff member position.

Of the responding departments, 44% prefers to appoint a radiologist with extra training in one or more subspecialties. The top three of most desired subspecialties is: musculoskeletal imaging, interventional radiology and breast imaging.

**Conclusion:**

Half of the responding graduates did not find a permanent staff member position before graduation. However, >90% found such a position within the first two years after graduation. There is a demand for dedicated profiles in almost half of the radiology departments.

## Introduction

There are 1888 radiologists in Belgium and the number is slightly increasing each year. Belgium has an average of 16 radiologists per 100,000 inhabitants, which is slightly more than the European average of 12.7 [1,2]. One would thus expect that a high number of newly graduated radiologists is required every year to replace the retiring ones, which is in contrast with the feedback we received from young and graduating radiologists who are having or have experienced difficulties finding employment. A survey was done in 2015, revealing difficulties in employment for young radiologists and an increasing demand for highly dedicated profiles in non-university hospitals [3].

How easy or difficult is it for graduating and recently graduated radiologists to find a job? Did they do an extra training in Belgium or abroad before finding a permanent job as a radiology staff member? How long did they have to search for this permanent position? These are only a few of the questions we wanted to elaborate.

We could not survey the young radiologists without listening to the heads of the radiology departments. What is their view on future job opportunities in radiology? Which external factors influence their decision to hire a new staff member? Do they expect to hire an increasing or decreasing number of new radiologists in the future compared to the previous years?

To answer those questions, the Young Radiologist Section (YRS) of the Belgian Society of Radiology (BSR) organized in the summer and fall of 2018, a survey on opportunities and difficulties in current and future employment for radiologists in Belgium.

## Material and methods

With approval of the board of the BSR, we started a survey using an online platform (Question Pro) from June 2018 until November 2018. In order to achieve a high response rate, our surveys were short and completely anonymous. To ensure anonymity, specific questions about the exact number of staff members or specific locations of hospitals in Flanders, Brussels or Wallonia were omitted. All surveys consisted of maximum ten multiple choice questions. The duration to fill out the surveys was less than five minutes per survey.

Two groups of recently graduated radiologists were surveyed: those who graduated in 2018 and those who graduated from 2013 until 2017. They were invited using e-mail addresses we obtained from the BSR and from the different Belgian universities. The heads of the departments, university and non-university (including private practices), were invited using e-mail addresses we obtained from the BSR database. After sending an initial invitation and three reminders by e-mail, a printed copy of the survey was sent to the different radiology departments in Belgium.

## Results

The highest response rate was achieved in the group of heads of university departments and the lowest response rate in the group of graduates 2013–2017. An overview of the response rates of the different study groups is shown in Table 1. An overview of the results in the different groups that were surveyed, is displayed in Tables 2 and 5.

**Table 1:** Response rates of the surveyed groups.

Surveyed Group	Number of invitations	Number of responders	Response Rate

Graduates 2018	46	23	50%
Graduates 2013–2018	225	89	40%
Heads of non-university departments	84	39	46%
Heads of university departments	7	5	71%

**Table 2:** Results of the survey of the radiologists graduated in 2018. UA: Universiteit Antwerpen. VUB: Vrije Universiteit Brussel, UGent: Universiteit Gent, KUL: Katholieke Universiteit Leuven, UCL: Université Catholique Louvain, ULC: Université Libre Bruxelles, ULiège, Université de Liège.

	UA	VUB	UGENT	ULIÈGE	UCL	ULB	KUL

AT WHICH UNIVERSITY HAVE YOU JUST GRADUATED?	8.7%	8.7%	17.4%	17.4%	17.4%	4.4%	26.0%

	I have started as a staff member in a radiology association	I have found a position as a staff member, but I am doing a fellowship or extra training before I start there	I am doing a fellowship while searching for a staff member position	I am unemployed for the moment	Other		
What kind of job have you started since your graduation	50%	8.3%	37.5%	4.2%	0%		

	Yes	No	No preference				
If you have found a permanent position in an association, is this in the region you initially wanted to work?	73%	14 %	13%				

	University Hospital	Non-University Hospital	Private practice	Hospital abroad	Other		
If you have found a job as a staff member, is this in a:	27%	52%	7%	7%	7%		

	University Hospital	Non-University Hospital	Other				
If you are doing a fellowship, is this in a:	27.3%	63.6%	9.1%				

### Graduates 2018

Our global response rate was 50% (n = 23) (Table 1), more Flemish (n = 14) than Walloon (n = 9) residents participated in the survey (Table 2). The survey was performed from July to August 2018, during the last months of residency of these graduates.

**Figure 1:**
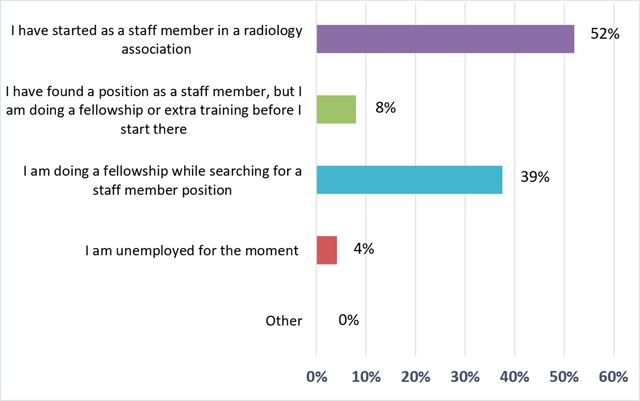
‘What kind of job will you start or have you started since your graduation?’ Results from the survey sent to the graduates of 2018.

**Figure 2:**
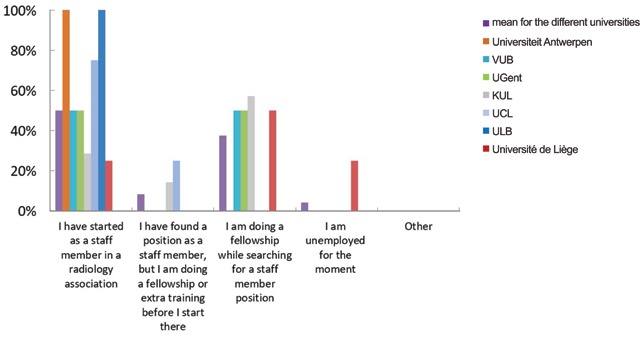
‘What kind of job will you start or have you started since your graduation?’ Results from the survey sent to the graduates of 2018, distributed per university.

At the time of the survey, 50% (n = 12) of the participants had found a position as a staff member in a radiology association starting shortly after graduation and 8% (n = 2) had found a permanent position at that moment, but with additional training/fellowship beforehand. A significant percentage (39%; n = 9) has started or planned a fellowship out of necessity, due to lack of finding a permanent position and 4% (n = 1) did not have a prospect of a temporary or permanent job (Figure 1). In the French speaking part of the country, a higher percentage (66%; n = 6) of the respondents had found work before graduation compared to the Dutch speaking part (57%; n = 8) (Figure 2). However, due to the small number of respondents in this survey, we can’t draw any conclusions from these percentages.

**Figure 3:**
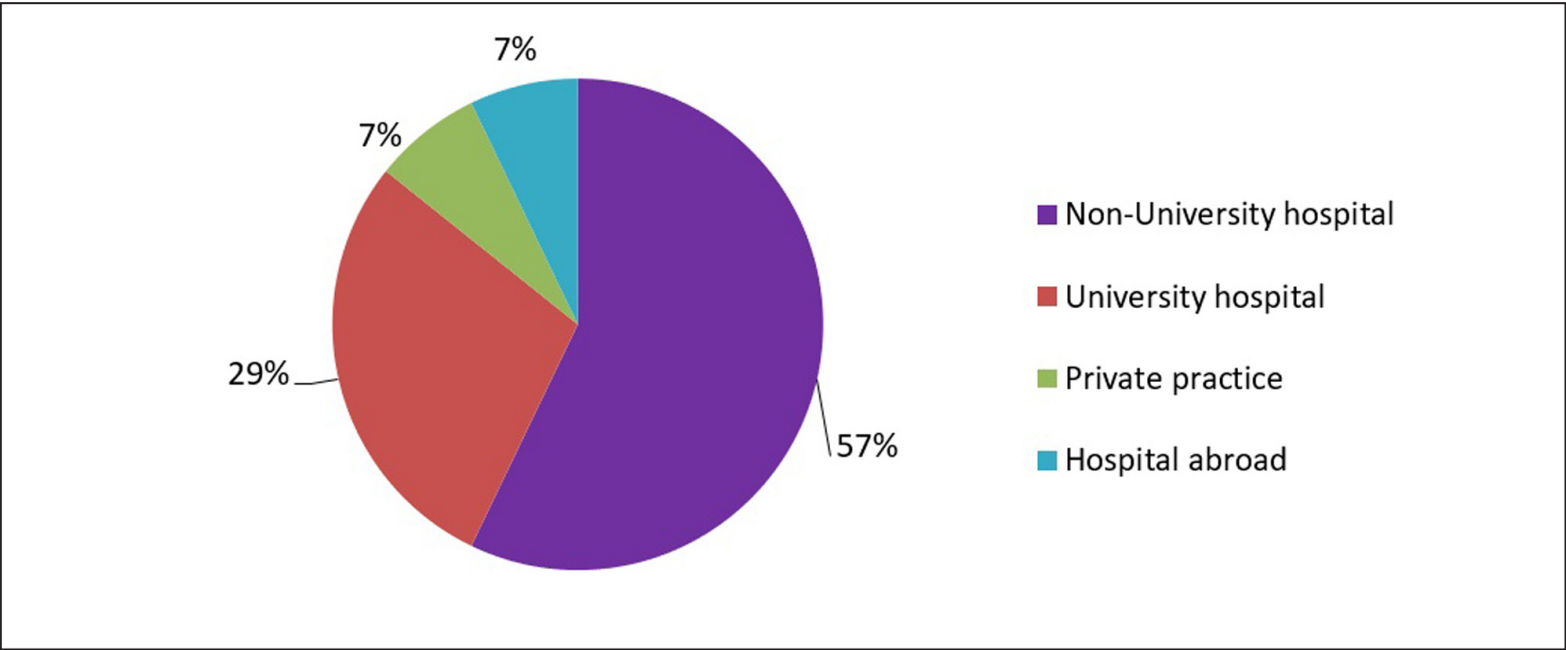
‘If you have found a job as a staff member, where is this?’ Results from the survey sent to the graduates of 2018.

Of the respondents who had found a permanent position (n = 14), 57% (n = 8) were in a non-university hospital, 27% (n = 4) in a university hospital, 7% (n = 1) in a private practice and 7% (n = 1) in a hospital abroad (Figure 3).

We analysed the types of fellowships graduates started: 27% (n = 3) of the respondents who started a fellowship after graduation, did this in an university hospital, 64% (n = 7) in a non-university hospital and 9% (n = 1) abroad. Remarkably, in Wallonia only 1 out of the 4 persons who started a fellowship did this in a non-university hospital.

Of the respondents who had already found a permanent position at the time of the survey, 77% (n = 10) had found a job in the region they initially wanted to work, while 15 % (n = 2) were working in a different region than their preferred. Fifteen percent (n = 2) didn’t have a preference.

### Graduates 2013–2017

A response rate of 40% (89/225) was achieved (Table 1). Of all the respondents, 27% (n = 24) were from the French speaking universities and 73% (n = 75) were from the Dutch speaking universities (Table 3).

**Table 3:** Results of the survey of the radiologists graduated in 2013 to 2017. UA: Universiteit Antwerpen. VUB: Vrije Universiteit Brussel, UGent: Universiteit Gent, KUL: Katholieke Universiteit Leuven, UCL: Université Catholique Louvain, ULC: Université Libre Bruxelles, ULiège, Université de Liège.

	UA	VUB	UGENT	KUL	UCL	ULB	ULIÈGE

At which university did you graduate as a radiologist?	7.9%	11.2%	11.2%	42.7%	10.1%	14.6%	2.3%

	2013	2014	2015	2016	2017		
In what year did you graduate as a radiologist?	9.0%	23.6%	16.9%	24.7%	25.8%		

	No	Yes, in a Belgian university hospital	Yes, in a non-university hospital	Yes, a fellowship abroad	Other		
Did you do an extra fellowship after general radiology?	41.8%	17.4 %	17.4%	21.4%	2.0%		

	To further specialize	Out of necessity	With prospect of a permanent position	Other			
What was the main reason to do this extra training?	43.9%	29.8%	10.5%	15.8%			

	Before my graduation	<6 months after graduation	Between 6 months and 1 year	1–2 years	2–3 years	>3 years	Not yet
How long after your graduation did you find a ‘permanent’ position?	57.3%	5.6%	10.1%	9.0%	6.7%	1.1%	10.1%

	Yes	No	I had no preference				
If your have found a permanent position, is this in the region you initially intended to work?	59.8%	24.4%	15.9%				

	University Hospital	Non-University hospital	Private practice	Hospital abroad	Other		
If you are currently working as a staff member, this is in a:	36.1%	56.6%	2.4%	2.4%	2.4%		

**Figure 4:**
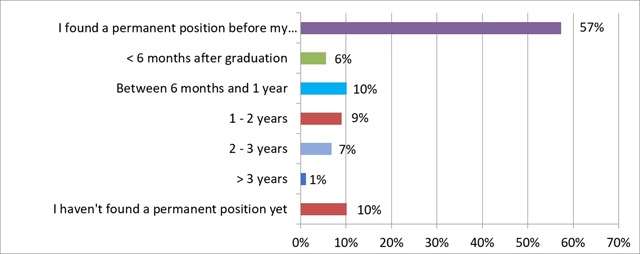
‘How long after your graduation did you find a ‘permanent’ position?’ Results from the survey sent to the graduates of 2013–2017.

More than half of the respondents (57%; n = 51) found a job before graduation. Another 16% (n = 14) found a permanent job within two years after graduation. A small percentage of the respondents who have found a permanent job (8%; n = 7) needed more than two years to find it. Of all the respondents, 10% (n = 9) are still looking for a permanent position and 7% (n = 6) have been searching for more than two years. These results are depicted in Figure 4.

**Figure 5:**
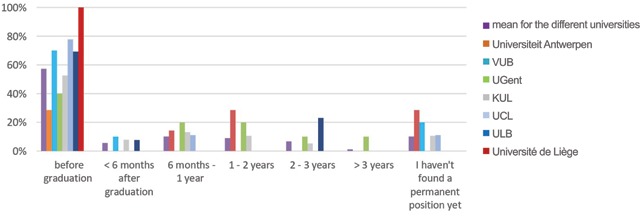
‘How long after your graduation did you find a ‘permanent’ position?’ Results from the survey sent to the graduates of 2013–2017, distributed per university.

A higher percentage of French speaking graduates (75%; n = 18) found a permanent job before graduation, compared to the Dutch speaking graduates (44%; n = 33). This is in line with the results in the group of graduates of 2018 (Figure 5).

**Figure 6:**
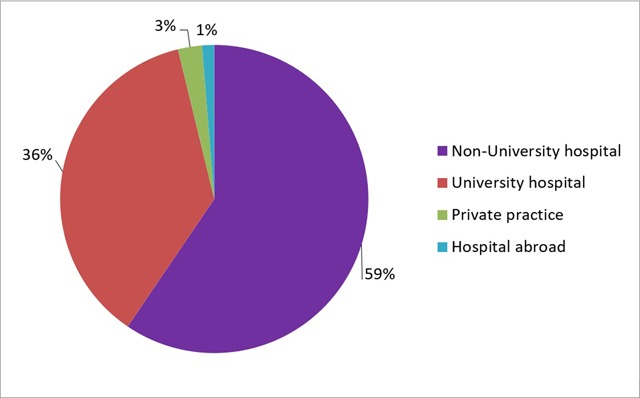
‘If you are currently working as a staff member, where is this?’ Results from the survey sent to the graduates of 2013–2017.

Of the respondents who have found a position as a staff member, 59% (n = 47) are working in a non-university hospital, 36% (n = 29) in a university hospital, 3% (n = 2) in a private practice, 1% (n = 1) in a hospital abroad and 1% (n = 1) did not specify his or her employment situation (Figure 6).

Of the respondents who have found a permanent position, 61% (n = 49) is working in the region they initially wanted to work, 25% (n = 20) in a different region than their preferred, and 14% (n = 11) did not have a preference.

**Figure 7:**
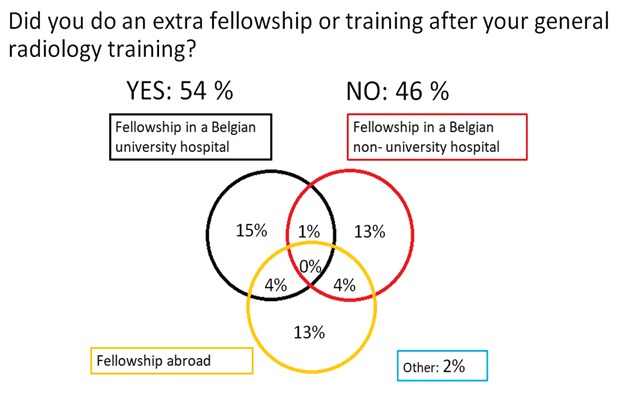
‘If you have done or are doing a fellowship, where is this?’ Results from the survey sent to the graduates of 2013–2017.

Fifty-four percent (n = 48) of the interrogated persons have done or were doing an extra fellowship/training after their general radiology residency. The distribution of the fellowships is displayed in Figure 7. Worth mentioning is that none of the French speaking respondents did a fellowship in a Belgian non-university hospital.

**Figure 8:**
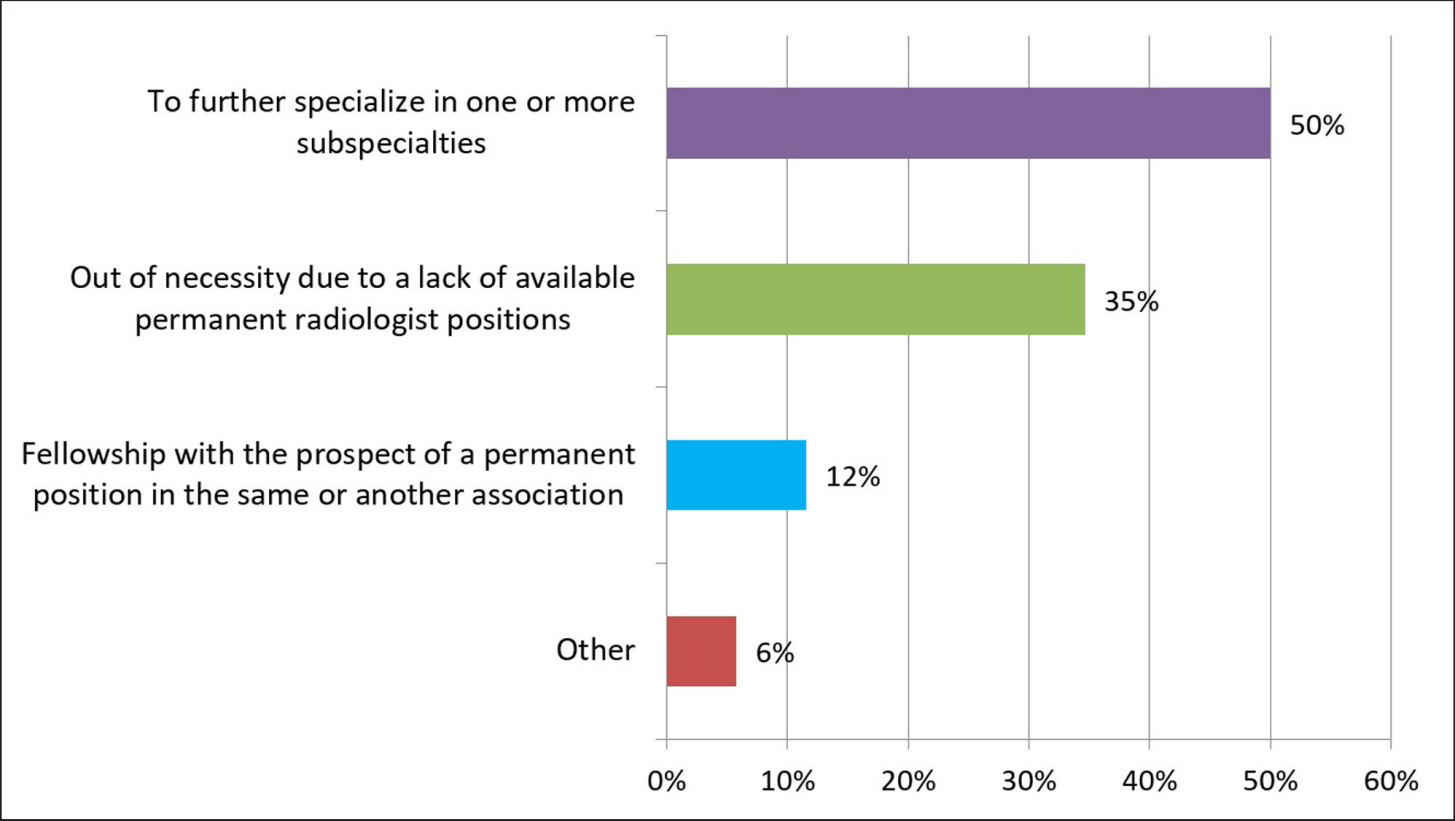
‘What was the main reason to do this extra training?’ Results from the survey sent to the graduates of 2013–2017.

When asked about the main reasons to do such a fellowship, 50% (n = 24) did this to further specialize in one or more subspecialties, 35% (n = 17) out of necessity due to a lack of available permanent positions and 12% (n = 6) with the prospect of a permanent position (Figure 8).

### Heads of the university radiology departments

In this small group, the response rate was high (71%; n = 5) (Table 1). Sixty percent (n = 3) of the responding heads of the University departments was looking for a new staff member at the time of the survey and highest in demand were subspecialists in musculoskeletal, cardiac and head and neck imaging (Table 4).

**Table 4:** Results of the survey of the heads of departments of the university hospitals.

	None	1–3	4–6	7–9	>9

How many vacancies for staff members were filled in your association in since january 2013?	0%	0%	40%	40%	20%

	Yes	No			
Are you currently searching for a new staff member?	60%	40%			

	Choice 1	Choice 2	Choice 3		
Which profile would fit best in your association?	Musculo-skeletal imaging	Cardiac imaging	Head and neck imaging		

	None	1–3	4–6	7–9	More than 9
How many staff members do you plan to hire in the next five and a half years (until december 2023)?	0	20%	60%	20%	0%

### Heads of the non-university departments

We achieved a response rate of 46% (n = 39) in this group (Table 1). Most of our responding radiology facilities (69%; n = 27) are from the Dutch-speaking part of the country and are medium-sized (51%; n = 20), ranging from five to eight radiologists (Table 5). About half of the responding departments (51%; n = 20) had radiologists-in-training and 26% (n = 10) was employing one or more temporary work force(s) (non-staff member graduated radiologists or ‘fellows’) at the time of the survey (Table 5).

**Table 5:** Results of the survey of the heads of departments of the non-university hospitals.

	Flanders	Brussels	Wallonia			

Where is your hospital located?	69.3%	5.1%	25.6%			

	1–4	5–8	9–12	13–16	17–20	>20
How many full time equivalent staff members does your association count?	5.1%	51.3%	15.4%	20.5%	0%	7.7%

	None	1	2	3	4	>4
How many vacancies for staff members were filled in since january 2013?	0%	30.8%	28.2%	15.3%	23.1%	2.6%

	General radiologist after training at one of the Belgian universities	Radiologist with an extra training in Belgium or abroad	A radiologist with some (>3) years of experience	Inter-national expert in radiology	A radiologist with extra competences in hospital management.	Other
Which profile would fit best in your association?	29.6%	43.8%	7.8%	9.4%	7.8%	1.6%

	Choice 1	Choice 2	Choice 3			
If you would employ a new radiologist, which subpecialty would you prefer? (multiple anwers)	Musculoskeletal imaging (17.0% of responders)	Vascular and interventinal radiology (14.0% of responders)	Breast imaging (12.0% of responders)			

	None	1	2	3	>3	
How many graduated temporary work forces were employed in your association in 2017?	74.4%	10.3%	5.1%	5.1%	5.1%	

	Yes	No				
Are there currently radiologists-in-training in your association?	51.3%	48.7%				

	None	1	2	3	>3	
How many staff member vacancies do you estimate to become available until december 2023	12.1%	30.3%	36.4%	18.2%	3.0%	

	Expected cooperations between hospitals	Expected reforms in the financing of the health care system.	The availability of temporary work forces	Retirement of an actual staff member.	Other	
Which of the following elements influence your decision to hire a new staff member?	24.7%	23.3%	7.8%	40.3%	3.9%	

**Figure 9:**
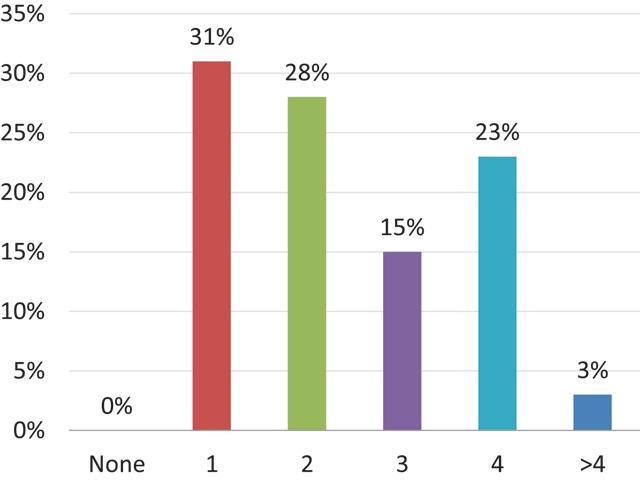
‘How many staff member vacancies were filled in since January 2013?’ Results from the survey sent to the heads of the non-university radiology departments.

**Figure 10:**
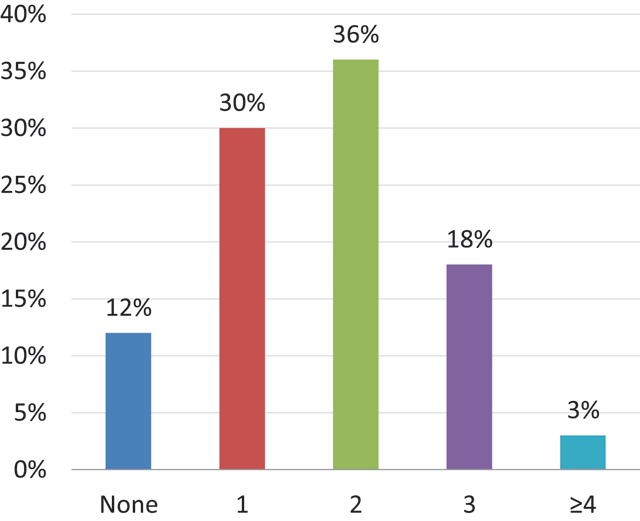
‘How many staff member vacancies do you estimate to become available from now until December 2013?’ Results from the survey sent to the heads of the non-university radiology departments.

All the respondents had filled in a staff member vacancy in their department within the last five years (Figure 9). When asked about the future, 12% (n = 4) thought they will not fill in new vacancies during the following five years (Figure 10). In the previous five years, 26% (n = 10) had hired four or more radiologists (Figure 9), whereas only 3% (n = 1) estimates they will need four or more new radiologists in the next five years (Figure 10). However, these data are not strictly comparable: the first question is a quite objective measurement, whereas the second question reflects more the sentiment of the head of the department at a certain moment of time.

**Figure 11:**
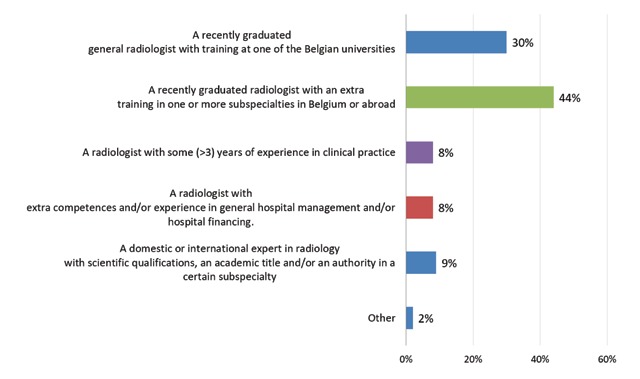
‘If you would employ a new radiologist, which subspecialty would you prefer?’ Results from the survey sent to the heads of the non-university radiology departments.

**Figure 12:**
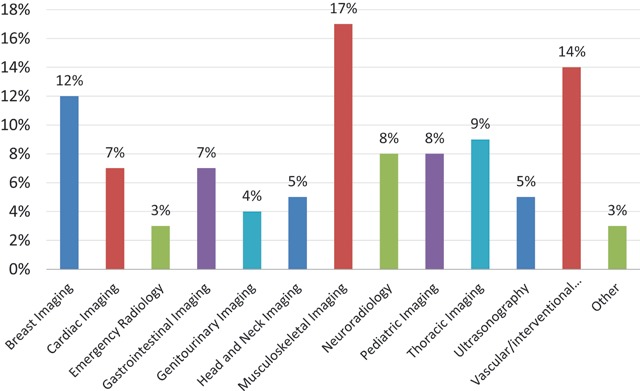
‘If you would employ a new radiologist, which subspecialty would you prefer?’ Results from the survey sent to the heads of the non-university radiology departments.

Of the responding departments, 44% (n = 17) would prefer a radiologist with extra training in one or more subspecialties when hiring a new colleague (Figure 11). The top three of most desired subspecialties were: musculoskeletal imaging, vascular and interventional radiology and breast imaging (Figure 12).

**Figure 13:**
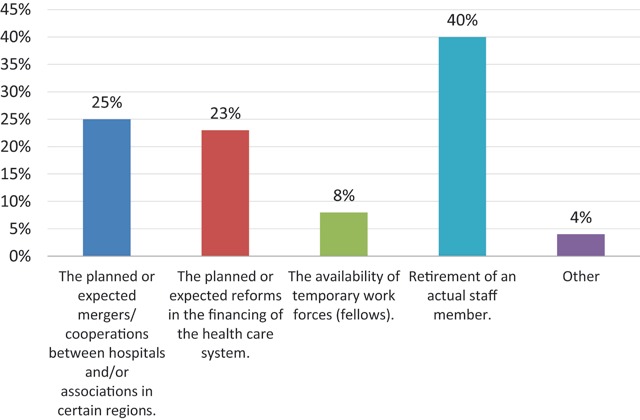
‘Which of the following elements influence your decision to hire a new staff member?’ Results from the survey sent to the heads of the non-university radiology departments.

We also asked about factors that influence the radiology departments in their decision to hire a new staff member. The most frequent answers were: retirement of a staff member (n = 31), current and upcoming changes in hospital networking (n = 19) as well as reforms in financing of the healthcare system (n = 18) (Figure 13).

## Discussion

To our knowledge, this is the first time a survey of the Belgian radiology job market is performed with focus on the recently graduated radiologists as well as on the heads of radiology departments. Due to the study design and the rather variable response rates, we cannot provide conclusive evidence that the radiology job market in Belgium is over- or undersupplied. This question has already been investigated in multiple countries which different results [4,5,6]. Given that is difficult to exactly map the needs of the job market, it can be said that it is not so easy for policy-makers to match the inflow of new radiologists to the job market.

Before further arguments, we must emphasize that despite all efforts with the available means, the highest achieved response rate was 50% in the groups of graduates from 2018. Only in the small group of heads of university departments, we obtained a higher response rate of 78%. As for every survey, this survey was susceptible to responder bias. One obvious responder bias was the high percentage of graduated radiologists who started working at university hospitals. We assume this is partly due to the fact that these people often kept the same e-mail addresses and were thus easiest to contact. On the contrary, people who started working in a different hospital after graduation often changed their e-mail address used for correspondence and therefore were more difficult to contact.

### Graduates 2018 and Graduates 2013–2017

Regarding the responses of the graduated radiologists, it is noteworthy that in both surveyed groups (graduates in 2018 and in the period of 2013–2017), about half of the respondents had found a permanent position before graduation. In both interrogated groups this percentage is higher in Wallonia than in Flanders. Approximately a third of the graduates is working as a staff member in a university hospital, however this result is probably skewed due to responder bias. For a third of the graduates who did or is doing a fellowship, the main reason for this was the inability to find a permanent job. Fellowships are being done more or less equally in hospitals abroad, university hospitals in Belgium and non-university hospitals in Belgium. However, Dutch speaking radiologists are doing their fellowships predominantly in non-university Belgian hospitals or abroad, whereas the French speaking radiologists are doing their fellowships primarily in university hospitals or abroad.

### Heads of departments

One logical limitation of this study is the inability to provide an exact number of radiology vacancies that will become available in the following years. Nevertheless, a close look to this survey’s results reveals a slight tendency towards a lower degree of employment in the coming five years.

Almost half of the responding non-university departments prefer a recently graduated radiologist with extra training in one or more subspecialties as their new colleague. Interventional radiology, musculoskeletal imaging and breast imaging are the most desired subspecialties. Research on subspecialty preferences has also been conducted in the United States and, strikingly enough, breast imaging and musculoskeletal imaging were also present in the top 3; on the other hand neuroradiology was ranked third place [7].

One surprising final result: recent and expected changes in hospital networking and health care financing are determining factors for almost one quarter of the responding departments in their decision to hire a new staff member.

### Final note

The issue of job opportunities in radiology is certainly an actual issue in Belgium, with the recent publication of the Royal Degree of 12 June 2018 in the Belgian statute book (Belgisch Staatsblad/Moniteur Belge) which formulated federal guiding quota for the number of residents accepted into radiology training. These guiding quotas, applying from 2023, will limit the number of Belgian residents able to start their radiology training to the number of 25 per year. The division will be as follows: 10 in the French speaking universities and 15 in the Dutch speaking universities. Nowadays, there are no fixed or guiding quota, but approximately 45 radiology residents start their training every year in Belgium (+/– 25 in the Dutch speaking universities and +/– 20 in the French speaking universities). Finally, it is worth mentioning that in 2023 an exceptionally large number of radiology residents will be graduating, due to the double cohort of radiology residents that have started their training in 2018 (a direct consequence of the switch from a seven to six-year programme in medicine studies in Belgium).

## Conclusion

A significant percentage of the responding graduates (around 50%) did not find a permanent staff member position before graduation. However, >90% found such a position within the first two years after graduation. There is a demand for dedicated profiles in almost half of the radiology departments.
